# Clinical inertia is the enemy of therapeutic success in the management of diabetes and its complications: a narrative literature review

**DOI:** 10.1186/s13098-020-00559-7

**Published:** 2020-06-17

**Authors:** F. Andreozzi, R. Candido, S. Corrao, R. Fornengo, A. Giancaterini, P. Ponzani, M. C. Ponziani, F. Tuccinardi, D. Mannino

**Affiliations:** 1 Department of Medical and Surgical Sciences, University Magna Graecia of Catanzaro, Viale Europa, 88100 Catanzaro, Italy; 2 Diabetes Center District 3, Azienda Sanitaria Universitaria Integrata di Trieste, Trieste, Italy; 3 Department of Internal Medicine, University of Palermo, Palermo, Italy; 4 SSD of Diabetology and Metabolic Diseases, Hospital of Chivasso, Turin, Italy; 5 Diabetology Service, Muggiò Polyambulatory, Monza, Italy; 6 Operative Unit of Diabetology, “La Colletta” Hospital, Genoa, Italy; 7 SSD of Diabetology-Azienda Sanitaria Locale Novara, Novara, Italy; 8 Diabetology and Endocrinology Unit “Clinica del Sole” Formia, Formia, Italy; 9 Section of Endocrinology and Diabetes, Bianchi Melacrino Morelli Hospital, Reggio Calabria, Italy

**Keywords:** Clinical inertia, Diabetes care, Italian association of medical diabetologists, Therapeutic inertia, Type 2 diabetes mellitus

## Abstract

Diabetes mellitus is a chronic disease characterized by high social, economic and health burden, mostly due to the high incidence and morbidity of diabetes complications. Numerous studies have shown that optimizing metabolic control may reduce the risk of micro and macrovascular complications related to the disease, and the algorithms suggest that an appropriate and timely step of care intensification should be proposed after 3 months from the failure to achieve metabolic goals. Nonetheless, many population studies show that glycemic control in diabetic patients is often inadequate. The phenomenon of clinical inertia in diabetology, defined as the failure to start a therapy or its intensification/de-intensification when appropriate, has been studied for almost 20 years, and it is not limited to diabetes care, but also affects other specialties. In the present manuscript, we have documented the issue of inertia in its complexity, assessing its dimensions, its epidemiological weight, and its burden over the effectiveness of care. Our main goal is the identification of the causes of clinical inertia in diabetology, and the quantification of its social and health-related consequences through the adoption of appropriate indicators, in an effort to advance possible solutions and proposals to fight and possibly overcome clinical inertia, thus improving health outcomes and quality of care.

## Key Summary Points

- The phenomenon of clinical inertia is defined as the failure to start a therapy or its intensification/non-intensification when appropriate, in diabetology.
- Despite the introduction of many glucose-lowering therapies that have proved to be efficacious in clinical trials, glycaemic control remains suboptimal in many patients globally, at all stages of treatment intensification.
- There is a clear need to encourage earlier intensification and address issues around therapeutic inertia to make health systems more sustainable and improve the quality of life of diabetic patients.
- Three classes of factors emerge as causes of clinical inertia: factors related to the healthcare professionals, to the patients and to the National Healthcare System.
- Through adequate training, clinicians can evaluate their own performances, identify critical areas and adopt suitable strategies, in a virtuous quality cycle able to increase knowledge, and modify behaviors.
- A structured and continuous educational activity, able to improve patients’ self-management abilities and responsibility, is fundamental.
- The creation of multi-professional teams able to work with a common and shared language, the planning of educational activities, the presence and implementation of specific and shared diagnostic-therapeutic paths, the creation of pathology registers, and the evaluation of performances with the use of indicators, are all plausibly effective organizational strategies to improve the clinical-care outcomes and reduce clinical inertia.

Background Diabetes mellitus is a chronic degenerative disease characterized by high risk of complications and high social, economic and health burden. To date it is a public health problem, as the incidence and prevalence of diabetes are constantly increasing worldwide, particularly in developing countries. Globally, in 2017, people with type 2 diabetes were about 425 million and it has been estimated that in 2045 there will be about 629 million diabetics [1]. The main concern about this epidemics is the growing number of people who develop diabetes-related complications [2, 3].

Numerous clinical studies have shown that optimizing metabolic control in patients with diabetes may reduce the risk of micro and macrovascular complications related to the disease. The UKPDS Post Trial Monitoring Study, comprising 5102 patients, showed that intensive control of glycated hemoglobin (HbA1c) from the time of diagnosis can reduce the risk of myocardial infarction, and mortality in general [4]. These data are also supported by the results of the ADVANCE and VADT studies in which patients on intensive treatment who reached lower HbA1c values had lower risk of developing both micro and macrovascular complications [5–7]. The STENO2 study further confirmed the superiority of glycemic control via intensive intervention with respect to the traditional approach, in preventing cardiovascular complications and reducing the risk of fatal and non-fatal events [8–11].

Approximately 20 years have passed since the UKPDS report, and the current Guidelines and Recommendations adopted by the various international and national Scientific Societies advocate for personalized but stringent metabolic targets, also suggesting pharmacological intervention algorithms to guide and facilitate their achievement. In all these documents, the metabolic targets are always identified based on patient’s characteristics, and the algorithms expand this concept taking into account not only the glycemic target, but also comorbidities and cardio-nephro-vascular risk profile of the patients.

Despite these excellent efforts, the phenomenon of clinical inertia (also termed therapeutic inertia) is an undeniable reality of diabetology (Boxes 1 and 2), studied for almost 20 years, since when, in 2001, Phillips et al. coined this term to indicate the failure to start a therapy or its intensification/de-intensification when appropriate, in diabetology [12]. Many population studies have observed that recommendations and guidelines are not adequately implemented in the clinical practice and glycemic control in diabetic patients is often inadequate (Box 3).

**Box 1—Defining clinical inertia in diabetes care**

The discrepancy between Guidelines and clinical practice is defined in the literature as “clinical inertia” or “therapeutic inertia”. Although these terms are usually employed in diabetology to indicate the lack of insulin initiation, according to Khunti and Davies [13] the concept of inertia can be extended throughout the natural history of diabetes for any lack of intervention that could lead to:

- primordial prevention (normal glucose tolerance), able to reduce the percentage of people at risk of diabetes;
- primary prevention (IFG/IGT), capable of reducing the percentage of people with IGT or IFG who progress to diabetes by up to 30% [14–18]:
- secondary prevention (clinical diabetes);
- tertiary prevention that identifies complications in their early stages.

It is therefore possible to define inertia as every instance in which no action is taken to promptly address each of these phases [13]. To avoid the risk of erroneously consider inertia a good practice (defined as “apparent” inertia) for a specific patient or clinical condition for which the guidelines do not provide definitive answers or there is no robust evidence in the literature [19–22], a new definition has been recently proposed, able to label clinical inertia if the following conditions are verified [23–25]:

- implicit or explicit guidelines exist;
- the doctor is aware of the guidelines;
- the doctor believes that the guidelines apply to the patient;
- the doctor has the resources to apply the guidelines;
- all these conditions have been met, but the doctor does not apply the guidelines to the patient.

**Box 2—The other face of inertia**

When talking about therapeutic inertia, overtreatment (usually of elderly people) and “failure to de-intensify diabetes therapy” cannot be overlooked, since they constitute “the other face” of inertia and a large problem in diabetes care [26]. Overtreatment is defined by the Institute of Medicine as the use of a treatment even when the potential harms exceed the possible benefits [27]. In older adults with diabetes, the harm of intensive glycaemic control likely exceeds the benefits [28–31]. In patients with high clinical complexity as well, intensive treatment significantly increases the risk of severe hypoglycaemia [32]. Applying the principles of evidence-based medicine (EBM) to the clinical decision making process is a key strategy that physicians can use both at the bedside and in guideline development and policy decisions, to prevent overtreatment [27]. This topic goes beyond the scopes of the present review, but it has been extensively studied and reviewed elsewhere.

**Box 3—Epidemiology of clinical inertia in diabetes care**

Inertia relating to diabetes management has been reported for over a decade with Shah and colleagues showing that less than half of a Canadian cohort of 2502 patients with type 2 diabetes and high HbA1c had received intensification of their treatment in 2005 [33]. In 2011 Fu and colleagues demonstrated a median time to intensification of treatment of 14 months in US clinical practice [34].

In 2012, the SOLVE study across North America, Europe and Asia (N > 17,000), documented that the average HbA1c reached 8.9% before insulin was initiated, and nearly half the patients had HbA1c ≥ 9.0% despite treatment with combinations of oral hypoglycemic agents [35].

In Europe, in 2013 and 2014, respectively, the GUIDANCE and PANORAMA studies reported that only 53.6% and 62.6% of patients achieved HbA1c ≤ 7% [36, 37]. More recently (2016), the GUIDANCE study showed that only 6.5% of patients had HbA1c > 9% [26], and in Germany most people with type 2 Diabetes had good glycemic control: 79% of patients under 70 years of age had HA1c ≤ 7% and 91% of those above 70 years had HbA1c ≤ 8% [38]. A further study performed in 2018 in Spain showed that therapeutic inertia was seen in 26.2% of patients with HbA1c > 7% and 18.1% of those with HbA1c > 8%, with issues of non-intensification occurring after a median follow up of 4.2 years [39]. In Italy, in 2011, patients with HbA1c < 7% were only 43.8%, in 2016 the percentage rose to 50.9%; in spite this improvement, in 2018 1 in 5 patients were still frankly unbalanced [40]. Independently of large regional variation, widespread delay of insulin initiation has been reported also in other Countries from Central and South-Eastern Europe [41, 42].

Finally, a retrospective cohort study investigating whether clinical inertia existed also in Japanese clinical practice, demonstrated that the estimated probability of intensifying treatment during the 12 months after recording HbA1c ≥ 7.0% (≥ 53.0 mmol/mol) was only 22.8%, and 27.5% after 17 months, evidence of clinical inertia in basal insulin-treated patients with type 2 diabetes in Japan [43].

The adoption of more “moderate” personalized objectives taking into account age and clinical fragility (i.e. comorbidities, life expectancy, duration of disease) does not justify the persistently poor metabolic control, nor the considerable proportion of people with HbA1c levels higher than 9.0%, as reported in many international studies [44]. The algorithms suggest that an appropriate and timely step of care intensification (introduction of a new drug or increase in dosages of ongoing therapies) should be proposed after 3 months from the failure to achieve metabolic goals [45]. A systematic review of 53 studies has recently shown instead that for above-target HbA1c levels, on average, a year can elapses before the intensification of therapy is implemented [46]. In addition to this, patients followed by primary care physicians appear to have more difficulty in achieving therapeutic goals due to delayed intensification, especially in patients on diet alone or in monotherapy [47, 48]. Consistent with this, data in the literature have revealed that, usually, diabetic patients achieve better glycemic control when they are followed by specialists [35, 49, 50], although a clear reason for this phenomenon has not been established. Possibly, diabetes care specialists are more aware of diabetological and cardio-vascular prevention aspects, they might offer more resources for the education of the patient, they can be more confident with prescriptions, therefore managing to be more “aggressive” in case of inadequate glycemic control [41–43, 51, 52].

Because clinical practice is often linked to highly complex situations, it is fundamental to distinguish true from apparent inertia, especially when trying to assess its causes and to identify solutions or improvement strategies. Indeed, it is difficult to establish when a therapeutic decision is appropriate for a particular patient without having information on the underlying clinical condition or intermediate traits.

Several studies clearly denounce the condition of “non-adherence” of the clinicians to the guidelines as a behavioural problem, because making therapeutic decisions is a complex task that involves a variety of cognitive processes. Indeed, uncertainty is one of the principal reasons that contribute to maintaining the status quo [53, 54]. Furthermore, the problem of decision-making delay does not only concern chronic diseases. Several studies about stroke and myocardial infarction have shown that much still needs to be done in order to improve the timeliness of intervention even in cases of emergency [55].

In the present manuscript, we will deal with the issue of inertia in its complexity, assessing its dimensions and its burden over the effectiveness of care. Our main goal is the identification of the causes of clinical inertia in diabetology, and the quantification of its social and health-related consequences through the adoption of appropriate indicators. In conclusion, we will try to advance possible solutions and proposals to overcome clinical inertia or at least reduce it, thus improving health outcomes and quality of care (Box 4).

**Box 4—How Can we Overcome the Barriers of Clinical Inertia?**

*Health professionals-related barriers* It is fundamental to identify the subjects at higher risk of delay in the intensification of the treatments [56]. Several studies have documented that active feedback to healthcare professionals and the introduction of specific informatic remainders are able to reduce the time of therapeutic intensification [57]. Proactive approaches with patients also prove useful, as patients respond better when they feel they contribute to a positive outcome [58]. The involvement of nurses, pharmacists, and other members of multidisciplinary teams in the management of the disease has proved effective [59] to help respond to patients’ needs and problems regarding their condition. Education end access to updated information on new drugs, including efficacy and adverse reactions, must be constantly available for healthcare professionals, as well as clear guidelines that can guide therapeutic choices.

*Patients-related barriers* On the patient side, educational interventions that make the person with diabetes fully aware and able to manage their condition represent a fundamental aspect. Telemedicine systems that allow healthcare workers to remotely monitor blood glucose values in the intervals between visits can guarantee continuity of care and reduce the time to therapeutic intensification. Nursing staff can help in case of poor compliance or anxiety associated with therapeutic problems, such as self-administration of injectable drugs [60]. Finally, it is possible to improve adherence to therapies through reminder systems and apps that support the patient in managing their diabetes [57].

*Healthcare System-related barriers* The National Health System should promote and facilitate chronicity management methods in line with technological advances, making use of telemedicine systems capable of guaranteeing the exchange of data and information between the healthcare facility and the patient [61]. Investing in innovative therapies, rather than an additional cost, could be an important source of savings, considering that the use of drugs capable of reducing major acute and chronic complications can have an important impact on spending, while improving patients’ life expectancy and quality of life [62]. A better organization of assistance, based on the real implementation of integrated care pathways would facilitate the continuity of care between primary and specialist care, making it easier and timelier to access diabetes services and prescribe innovative drugs, improving the appropriateness and adherence to guidelines based on scientific evidence.

This article reviews the current evidences concerning clinical inertia in patients with type 2 diabetes after an extensive research of the principal bibliographic citation databases like PubMed, Scopus and the Cochrane Central Register of Controlled Trials.

## Causes of clinical inertia in diabetes care

The causes of therapeutic inertia are multifactorial and complex and this phenomenon is becoming increasingly important in the management of diabetes also because it exposes patients to long periods of hyperglycemia which in turn foster an high risk of developing several complications and reduced life expectancy [63].

The causes of therapeutic inertia have been long debated, with the main goal of implementing strategies able to resolve and/or mitigate the problem.

From the careful analysis of the data currently available in the literature, three classes of factors have emerged as possible causes of clinical inertia, that is, factors related to the healthcare professionals, to the patients and to the National Healthcare System (Table 1). Almost all authors agree on the clinician’s greater responsibility as a cause of inertia [64]. Frequently, in fact, health professionals tend to delay the initiation and/or intensification of the treatment, in particular with insulin, because they are concerned that this procedure may entail clinical consequences and increased risk of hypoglycemic events, weight gain, difficulty in managing more complex injection therapies or at least the perception that the patient may have more difficulty in managing them. As for the patients, it is not uncommon for them to reject the doctor’s proposal to initiate or intensify insulin therapy, mainly because insulin therapy is perceived either as a “final stage” therapy, or as a punishment due to poor patient compliance [65]. These responsibilities will be examined extensively in the following section, bearing in mind, however, that if we want to try to solve the problem, it should be considered as a unique multifaceted phenomenon rather than a cluster of separate entities [66].

**Table 1:** Causes of clinical inertia

Clinician-related	Patient-related	Healthcare system/practice-related
Insufficient time	Denial of having the disease	No clinical guidelines
Work overload	Denial that the disease is serious	No disease register
Burn-out	Absence of symptoms	Bureaucratic difficulties with new drugs
Inadequate knowledge of Guidelines and up-to-date scientific evidence	Low health literacy	Inadequate technologies support
Lack of familiarity with the new therapies	Too many medications	Resource constraints
Failure to set clear goals	Therapeutic regimen too complex	Resistance to change in the system
Difficulty in managing more complex injection therapies	Medication side effects	No visit planning
Failure to initiate treatment	Depression or substances abuse	No active outreach to patients
Failure to titrate treatment to achieve goals	Lifestyle factors	No decision support
Fear of side effects	Cognitive, emotional and behavioral obstacles	No team approach to care
Difficulty in managing side effects	Poor communication between physician and patient	Poor communication between physicians and staff
Failure to identify and manage comorbidities (e.g. depression)	Psychological resistance to insulin	Not structured education activity
Reactive than proactive care		
Underestimation of patient’s need		
Inadequate physician–patient communication		
Presence of cognitive bias with lack of rationality in decision making		

### The barriers related to healthcare professionals (Table 1)

The barriers related to healthcare professionals include: lack of time, poor training, and lack of familiarity with the efficacy and safety of therapeutic regimens. These factors lead to an abuse of conventional drugs, such as metformin, sulfonylureas and insulin, therefore neglecting the new options of combined therapies or the new hypoglycemic molecules, either oral or injective, which present a window of efficacy and safety greater than the classic hypoglycemic agents [57].

Other important physician-related barriers are the recognition and management of side effects, the lack of awareness of the need to adopt a new therapeutic regime and the failure to establish and/or monitor all progresses achieved with respect to the set objectives. For this issue, the role of hypoglycemia is central: a study has shown that for 75.5% of healthcare professionals fear of hypoglycemia is a barrier to insulin therapy [67]. Zafar et al. also recognized other key factors, such as doctors’ misperception of improved glycemic control [68]. Parchman’s research team monitored 211 diabetological outpatient visits [69] and observed that the likelihood of a change in treatment decreased proportionally with the increasing degree of patient’s concern during the visit. This effect was independent from the duration of the visit, the value of HbA1c and its trend in time, the time elapsed since the previous evaluation, and the number of discussion points raised by the doctor.

One of the causes of clinical inertia, often declared by the same doctor, is the lack of awareness of the guidelines, which are frequently updated on the latest evidence resulting from clinical studies. The goals of treatment are not clear, the level of HbA1c to be achieved based on the patient’s characteristics is not known. Indeed, in recent years, based on scientific evidence, it has emerged that not all patients must reach a glycated hemoglobin level < 7% and this can lead to uncertainty in the healthcare professionals [70].

Equally important when talking about the physician’s responsibility as a cause of therapeutic inertia is their concern and/or conviction about certain patients compliance with the therapy, whom the doctor might perceive as incapable or reluctant to therapy changes and/or regimes ever more complex. Finally, one of the causes that is always declared by the doctor when it comes to therapeutic inertia is the heavy workload, often without an adequate organization and with high risk of burnout. In a chronic disease such as diabetes, where the patient is at the center of an articulated path focused on the recognition and satisfaction of clinical needs, the diabetologist is actually the main actor of the patient-centered approach. It is the diabetologist who must manage the therapeutic relationship with clinical, empathic, communication and organizational skills. Some neuroscientists have stated that clinicians often consider guidelines as treatment strategies based on clinical or experimental studies involving strict patient groups, which do not apply to particular patients (and each patient is particular) and with limited information. This increases the doubt in accepting the guidelines and generates overconfidence, aversion to risk or uncertainty, and herding [53]. Studies on decision-making theories by D. Kahneman have suggested that individuals, in the concrete act of making a decision, do not respond to optimizing logics but use a limited number of heuristics, or mental shortcuts. This could be attributed to the presence of cognitive bias, manifesting in situations of uncertainty [71]. For this reason, it is important to explore the conscious and unconscious mental processes at the base of the therapeutic choices in order to identify and highlight the factors related to therapeutic inertia.

### Patient-related factors (Table 1)

The patient-related factors that favor clinical inertia include drug side effects, inability to follow complex treatment regimens, poor awareness of the true severity of the disease, limited doctor-patient communication and low level of education, collectively accounting for about 30% of the cases of clinical inertia [23]. Furthermore, poor compliance with an adequate diet, socioeconomic status, presence of acute and terminal illnesses are barriers that can be difficult to overcome, but must be managed. Patients sometimes can feel discouraged and frustrated, and such feelings can push them to stop their medications, resulting in failure to reach the glycemic target [72].

The data from the PANORAMA study [Efficacy and Safety of Intravitreal (IVT) Aflibercept for the Improvement of Moderately Severe to Severe Nonproliferative Diabetic Retinopathy (NPDR)], carried out in France, showed that the HbA1c targets set by French doctors for their patients reflected a good knowledge of type 2 diabetes care guidelines. Nevertheless, over two-thirds of patients failed to reach their intended goal, and this issue was attributed to the reluctance of the patients to intensify their treatments [73]: a phenomenon defined as “psychological resistance to insulin”, present in about 25% of the population to which this drug was prescribed [74].

The patient’s perception of non-adherence may contribute to clinical inertia in intensifying oral hypoglycemic agents. According to an analysis carried out in the United States, in fact, the clinician is led to make changes in therapy (dosage or pharmaceutical formulation), in patients who show greater compliance [75]. It is also possible that the association between delayed treatment intensification and poor adherence, as reported by Grant [75], may represent a tactic to tackle the problem with adherence at first, and only subsequently proceed with the intensification of the treatment. However, the author confirmed an overall slower rate of intensification: even in the cohort with the highest adherence, intensification was delayed on average for 2 years. However, when the doctor believes that the patient might not have good compliance, it would be good clinical practice to not address the problem in a step-wise manner, but to discuss it with the patient in conjunction with the intensification of therapy.

Other factors inherent to specific treatments used for type 2 diabetes can also contribute to clinical inertia by affecting compliance. These factors are mainly related to the side effects of a treatment (hypoglycemia, weight gain, edema, gastrointestinal symptoms), to the perceived complexity of treatment administration or to poor efficacy of treatment on glycemic control [76].

Another reason why patients do not achieve their goals is called “educational inertia”, defined as the learning of clinically inaccurate or obsolete information by doctors and health professionals [77]. This misinformation is implemented in patient care, resulting in poor outcomes. Since educational inertia is a subjective concept, it is impossible to measure it. It would be desirable that, during congresses, conventions or annual events proposed by scientific associations, healthcare professionals be given the real information they need, to successfully help their patients and guide them in achieving personalized therapeutic goals [77].

### Factors related to the National Healthcare System (Table 1)

The evaluation of the barriers generated by the National Healthcare System cannot be generalized, because it variates according to the individual legislation and realities of each Country/Region. Among the numerous possible indicators of therapeutic inertia, a pivotal role is played by a poor coordination between planning and data exchange between the members of a health team, inadequate support technologies, the need for reimbursements, insurance coverage, and the great difference in regional norms [23]. The bureaucratic difficulties deriving from the use of therapeutic plans, for which expensive and complicated processes are required, lead doctors to adopt cheaper and more easily accessible drugs [78]. Resource constraints that limit staff time and predisposition to develop individual patient care plans can also limit the provision of in-depth diabetes education. The lack of an adequate care plan, including appropriate instructions on the use of medicines, can lead to delays in treatment intensification [64]. In situations where changes in health systems may be needed to improve care, the inertia of the system can make reform difficult. Clinical inertia can therefore be exacerbated by the inherent resistance to change within establishments facing barriers and competing demands [79]. More fundamental changes, such as a person-centered care model, can help find ways to address the challenges of patient non-compliance and clinical inertia.

## Use of indicators

The analysis of the literature does not clearly outline the indicators of therapeutic inertia, but it rather identifies the methods for measuring inertia itself. In particular, some authors measure it by evaluating the appropriateness of the care process in relation to reference standards (guidelines, care paths) and therefore through a methodology that can be equated with the use of process indicators (Table 2). In other instances, therapeutic inertia is measured by evaluating the effects on welfare outcomes, either clinical and economic (direct and indirect costs), through a methodology equated with the use of outcome indicators (Table 3).

**Table 2:** Process indicators employed in the literature to assess clinical inertia

	References
Percentage of therapy intensification in patients with HBA1c > 8% (addition of an oral hypoglycemic agent or dosage increase for an existing therapy or initiation of insulin treatment)	[33, 83]
Percentage of therapy intensification in patients with HBA1c > 7%	[84]
Percentage of initiation of insulin treatment in patients with HBa1c > 9%	[42]
Average time elapsed between type 2 diabetes diagnosis and initiation of insulin treatment in patients with non-target HBa1c	[42]
Percentage of patients with HBa1c > 7% undergoing basal insulin treatment for 180 days and subjected to the intensification of insulin therapy	[43]
Difference between the percentage of outpatient visits in which sBP was higher than the target minus the percentage of outpatient visits in which a modification of anti-hypertensive treatment was implemented, either type or dose of treatment, divided by the number of eligible visits. The resulting value is multiplied by the average difference between sBP as measured in all visits and the target value of sBP	[85]
Percentage of patients with non-target levels of LDL cholesterol and treated with statins, divided by the total number of eligible patients	[86]
Time (days) elapsed before a therapeutic intervention subsequent to sub-optimal lab test results	[87]
Percentage of healthcare professionals who prescribe the initiation of insulin therapy to patients with type 2 diabetes and HbA1c at the recommended threshold of 7–7.9%	[42]
Number of patients without therapy intensification, divided by the total number of patients with HbA1c ≥ 7%, multiplied by 100	[52]
Time spent with poor glycemic control (HbA1c 7%, > 7,5%, > 8%) in patients with type 2 diabetes treated with DPP-4i/SGLT-2i until the intensification of treatment with insulin/GLP-1RA	[88]
Percentage of patients lacking therapy intensification within 180 days from metformin failure	[89]

**Table 3:** Outcome indicators employed in the literature to assess clinical inertia

	References
Percentage of patients with HbA1c < 7%	[83]
Time required to reach targets of HbA1c, sBP and LDL cholesterol	[87]
Comparison between personalized HbA1c target and actual HbA1c levels	[73]
Percentage of patients who do not achieve the individualized targets	[90]
Life expectancy and economic burden associated with diabetes-related complications in populations reaching different targets of HbA1c, in a series of models of delayed therapy intensification e and across a range of time horizons	[91]
Median time to the progression of diabetic retinopathy	[92]
Incidence rate of diabetic retinopathy progression in presence or absence of clinical inertia (lack of initiation of insulin therapy within 3 months from a report of HBA1c > 9%)	[92]

In several cases the appropriateness of the care process has been evaluated as “failure to intensify the therapy in the presence of a clinical situation that made it necessary”. As part of diabetes therapy, intensification was intended as the addition of a new drug, the increase in the dosage of the existing therapy, or the initiation of insulin in the presence of non-target HBA1c values. As part of the intensification, failure to titrate the basal insulin after initiation was also included. This method of measuring inertia was also used for hypotensive and normolipemic therapy. In some instances, the time elapsed before intensification was evaluated, instead of the percentage of patients assigned to a specific care process.

The consequences of therapeutic inertia have been evaluated more frequently on clinical care outcomes as a percentage of target subjects for glycemic, lipid and blood pressure parameters or as time elapsed before clinical optimization. The impact of therapeutic inertia on more severe outcomes such as life expectancy and progression of retinopathy was also assessed. For the economic aspect, the focus was on the impact of therapeutic inertia on the costs of complications.

## Discussion and proposals to overcome clinical inertia

Therapeutic inertia, particularly in the management of chronic diseases such as diabetes, is a very complex phenomenon that recognizes multiple causes, largely dependent on the health professionals, but also on the patient and on national healthcare, with significant impact on health outcomes, welfare and social costs. In the past, the balance between hypoglycemia and strict metabolic control was difficult to assess with a limited therapeutic arsenal, and inertia was somewhat explained by fear of hypoglycemia. Nowadays, we have drugs with a very-low risk of hypoglycemia, able to minimize cardiovascular and renal burden. Therefore, we have entered an era where inertia is ethically unacceptable. In this manuscript we have analyzed the whole phenomenon, also trying to identify indicators to quantitatively and qualitatively measure inertia.

One of the possible approaches identified in the literature is to adopt monitoring systems to assess the quality of care as a whole. Through an analysis of process and outcome indicators and above all through a comparison among diverse care realities, clinicians can evaluate their own performances, identify critical areas and adopt suitable strategies, in a virtuous quality cycle. An attentive evaluation of behaviors and results could be instrumental for a real evolution of the entire class of professionals. From a practical point of view, it is crucial to implement discussions and comparisons in the various local realities to analyze the results obtained in each clinical practice and to make corrective actions.

Among the possible causes of therapeutic inertia, the main observations regarding health operators are the lack of familiarity with the new drugs, which are associated with a very-low risk of hypoglycemia and cardiovascular and renal events, the inadequate knowledge of Guidelines and up-to-date scientific evidences and the uncertainty about clinical objectives. From here it clearly emerges how training is fundamental to increase knowledge, but also for behavior modification: frontal readings, workshops and in-depth peer reviews, should be supplied alongside more accessible and interactive hands-on experiences combined with distance learning and evaluations.

Ideally, the therapeutic decision making could be guided by the implementation of software or algorithms embedded in the informatics clinical folders, able to stratify patients according to previous cardiovascular events, presence of chronic kidney disease, age, fragilities, etc., therefore identifying the most appropriate decision aligned with the current recommendations and candidates for new therapies [80].

Training courses at all levels of care (including general practitioners) and specialization (cardiologists, nephrologists, geriatricians, etc.) involved in the treatment of diabetes and its complications are essential to reach the greatest number of potentially treatable patients with new drugs since the early stages of illness. This, in addition to raising awareness among professionals and creating a common cultural base, would reassure patients who would receive uniform information shared by various professionals. Large-scale training projects might also help to overcome cognitive biases, indeed, it is necessary to identify the mental maps underlying therapeutic choices in order to recognize and reduce the inappropriate behavior [81].

Alongside a structured and continuous educational activity, able to improve patients’ self-management abilities and responsibility, all strategies able to increase adherence to therapy are important for antagonizing inertia: the simplification of therapeutic schemes, the preference for using drugs burdened by a lower impact on weight and hypoglycemic risk, the choice of therapy taking into account the patient’s preferences and lifestyle, sharing of the therapeutic objectives, the recognition of the cognitive, emotional and behavioral obstacles put in place by the patient as conscious and unconscious defenses to the therapy, an effective communication.

The clinical skills and up-to-date scientific knowledge of the professional must therefore be associated with relational, communicative and pedagogical skills. Recently, the skills required for a chronicity professional were examined and described in a Core Competence Curriculum, [82] through a correlation between activities/knowledge/skills and their impact on the Diabetology outcomes. The presence of an increasing number of professionals with “certified” skills is likely to foster a reduction of therapeutic inertia and an improvement in the quality of care and in the achievement of therapeutic goals.

Other effective organizational strategies to improve the clinical-care outcomes and reduce clinical inertia would be the creation of multi-professional teams able to work with a common and shared language, the planning of educational activities, the presence and implementation of specific and shared diagnostic-therapeutic paths, the creation of pathology registers, and the evaluation of performances with the use of indicators.

## Conclusion

In conclusion, only through a multifactorial approach able to affect all the elements at the basis of therapeutic inertia and through complex and complementary organizational, educational and training strategies, it will be possible to reduce this phenomenon and thus improve care outcomes, with a significant impact on health outcomes, on reduction of complications and health costs of diabetes.

## Data Availability

Not applicable.
